# Society Meetings

**Published:** 1900-06

**Authors:** 


### SOCIETY MEETINGS.

THE National Confederation of State Medical Examining and Licensing Boards will hold its tenth annual meeting in the banquet hall, Hotel Traymore, Atlantic City, N.J., Monday, June 4, 1900, under the presidency of Dr. J. N. McCormack, of Kentucky. The morning session will begin at 9.30 o'clock; the afternoon session at 2.30 o'clock.

The following program has been arranged: Invocation, Rev. Wm. Aikman, D.D., Atlantic City; Address of Welcome, Hon. F. P. Stoy, Mayor of Atlantic City; Address of welcome on behalf of the medical profession of Atlantic City, W. Blair Stewart; Address of welcome on behalf of the State Board of Medical Examiners, of New Jersey, Wm. Perry Watson, Newark; Response by Vice-president N. R. Coleman; Report of the Secretary-Treasurer; Annual address of the President—The work accomplished under the Kentucky law; Associate medical examining boards, Charles A. Groves, East Orange, N.J.; A brief review of the medical curriculum of the United States with special reference to its defects and indicated modifications, as demonstrated by the state medical examinations of Pennsylvania, Henry Beates, Jr., Philadelphia; The new medical law of Ohio, Hon. Maro J. Love, Bloomingville; The coöperation of the medical profession of the United States with the National Confederation of State Medical Examining and Licensing Boards in establishing interstate reciprocity for the license to practise medicine, Emil Amberg, Detroit, Mich.; Dicussion: What steps shall be taken to establish a uniform standard of preliminary requirements in accordance with the recommendations contained in the report of the committee on minimum standards, adopted June 5, 1899? The following members will lead, N. R. Coleman, Columbus, Ohio; William Warren Potter, Buffalo, N.Y.; Augustus Korndoerfer, Philadelphia, Pa.; Joseph M. Mathews, Louisville, Ky.

Miscellaneous business; election of officers; adjournment.

The following medical societies will hold their sessions in June, at Atlantic City, N. J.:

The American Academy of Medicine, June 2-4th.

The American Medical Association, June 5-8th.

Baltimore and Ohio Association of Railway Surgeons, June 1-2d.

American Medical Editors' Association, June 4th.

National Confederation of State Medical Examining Boards, June 4th.

Association of American Medical Colleges, June 4th.

American Association of Acting Assistant Surgeons, June 6th.

Medical Society of New Jersey, June 4th.

Confederation of State Boards of Health, June 1st-2d.

Railroad rates have been granted at a fare and a third, round trip, on the certificate plan, from all parts of the United States.

---

The Buffalo Academy of Medicine held meetings during the month of May, 1900, as follows:

*Section on Surgery.*—Tuesday evening, May 1st. Program: Fitting glasses on children, Arthur G. Bennett; Abscess of lung, Edward M. Dooley. Discussion by Drs. Mynter, Parmenter, E. A. Smith, Phelps and others.

*Section on Medicine.*—Tuesday evening, May 8th. Program: Report of case of influenza with four distinct pneumonic attacks.—Otitis media purulenta marasmus, with recovery, Julius Ullman. Typhlitis and scolicitis, A. L. Benedict. Dress reform, Madame Mankell.

*Section on Ophthalmology, Otology, Rhinology and Laryngology.*—Monday, May 14, Program: Report of cases of incubation of the larynx, Eugene A. Smith; Some notes on trachoma, E. E. Blaauw.

*Section on Pathology.*—Tuesday evening, May 15th. Program: Echinococcus cysts of the pleura, Charles Cary. Discussion by Irving P. Lyon. Gaseous disinfection, Dr. Pease; Syphilis, illustrated by stereopticon, Grover W. Wende.

*Section on Obsterics.*—Tuesday evening, May 22d. Program: Asepsis and anti-asepsis in obstetrics, D. W. Harrington; Obstetrical reminiscences, James S. Smith. Discussion of both papers by C. C. Wyckoff, Samuel G. Dorr, S. S. Greene, W. D. Greene and others.

---

The Physicians' Club, of Buffalo, held its regular monthly meeting, May 16, 1900, at the home of Dr. J. J. Finerty, 429 Franklin Street. The paper of the evening, entitled, "Cancer," was read by Dr. C. E.

Congdon, which will be published in the JOURNAL. Dr. J. Henry Dowd reported some cases in practice, after which an election of officers were held which resulted as follows: President, W. G. Ring; vice-president, W. C. Callanan; secretary-treasurer, D. P. Doyle; council, J. J. Walsh, D. J. Constantine, T. B. Carpenter, F. M. Boyle and James S. Porter.

